# Analysis of Sustainable Communication Patterns during the Telework Period in Western Romanian Corporations

**DOI:** 10.3390/ijerph19169796

**Published:** 2022-08-09

**Authors:** Eugenia Țigan, Radu Lucian Blaga, Florin-Lucian Isac, Monica Lungu, Ioana Anda Milin, Florin Tripa, Simona Gavrilaș

**Affiliations:** 1 Faculty of Food Engineering, Tourism and Environmental Protection, “Aurel Vlaicu” University of Arad, 2-4 Elena Drăgoi Str., 310330 Arad, Romania; 2 Faculty of Economics, “Aurel Vlaicu” University of Arad, 77 Revoluției Bdl., 310032 Arad, Romania; 3 Faculty of Management and Rural Tourism, Banat’s University of Agricultural Sciences and Veterinary Medicine “King Michael I of Romania” from Timisoara, Calea Aradului Street No. 119, 300645 Timisoara, Romania

**Keywords:** interpersonal communication, social and behavior change interaction, global companies, pandemic crisis, telework period

## Abstract

The research was conducted in a particular context, the recent pandemic. It is a comparative study of the methods and quality of communication in global companies between 2021 and 2022. The corporations involved in the research are important providers of flexible production, quality, and logistics solutions that cover customers’ real needs. They are active in the automotive industry and units involved in mass production in the electronics industry, household appliances, and cosmetics industries. In their case, it was noted that to achieve operational objectives such as developing employee skills, using advanced technologies, and exceeding customer expectations, it is important to use innovative methods and tools such as single platforms, which allow access to the most important information from a distance, anywhere, anytime. It is significant that, according to the research, the preferred method of communication by employees, regardless of the existing conditions, is face-to-face. Primarily, this method is chosen because it provides an open area of interpersonal interaction. The participants observe non-verbal attitudes or can perceive emotions and feelings. Their personality can be identified through unintentional contact to obtain constructive feedback through guidance and counseling. Moreover, it can be formed and develop productive, intentional connections. Stakeholders’ efficient and effective open dialogs are encouraged in this sense.

## 1. Introduction

This research was designed as a comparative study. It intended to capture and analyze two periods of global company activity in terms of communication methods preferred by employees at different organizational levels. The topic focused on determining the real situation regarding communicating during the recent pandemic. The possible negative influence on employees’ psychological health sustained the scientific approach [1]. Our interest was to establish if the situation had similar repercussions in different working fields. The first cases reported in the literature were for the healthcare domain, one of the areas most affected by the crisis [2,3]. Recently, the reference area was extended [4,5,6,7]. Encouraging memos positively influenced workers’ state of mind [8].

In this sense, our research was carried out in April–May 2021, and the second one year later. The corporations are located in the West Region of Romania, Arad, and Timiș counties. Their position is in the Western Development Region, one of the eight administrative regions of Romania (NUTS II-Nomenclature of Territorial Units for Statistics) [9].

To facilitate research and harmonize it with the concept of sustainability, the concern of today’s society is to achieve a balance between security and social cohesion, economic performance, and environmental protection. These aim to ensure the next generation’s future [10].

We must outline the region’s characteristics from a socio-economic perspective and the environment. This description was based on some of the indicators suggested by Sustainable Development Indicators at the Territorial level for Romania (SDIT) [11] and Structural Business Statistics (SBS) [12].

The West Region is located on the frontier with Hungary and the Republic of Serbia and borders Romania with the Central, North-West, and South-West Development Regions. The West Region has four component counties: Arad, Caraș-Severin, Hunedoara and Timiș (NUTS III) [13]. The region’s surface is 32,034 km^2^ (13.4% of Romania) and has a population of 2,003,368 inhabitants.

The evolution of the global participation rate for employees of companies in continuous professional training courses by size classes of enterprises in 2010–2015 is encouraging. In 2015, 21.3% of employees from all over the country participated in such activities, with 3.5% more than in 2010 [14]. The most concerned about the continuous training of employees in 2015 were large companies with a rate of 32.6% of total employees, medium-sized companies with a rate of 13.7%, and small ones with a participation rate of 8.8%.

The statistical data analysis indicates that in the West Region, there is a sharp imbalance in the distribution of companies at the regional/local level. Some areas (Arad, Timișoara) polarize significant SMEs (Small and Medium Enterprises). In contrast, in many areas of the region, the number of active companies is low (Caras-Severin, Hunedoara). The West Region is in seventh place regarding the number of companies, compared to the total national number, with a percentage of only 9% of the total of 624,206 companies in Romania in 2020 [15]. Very few domestic companies conduct business or export beyond Romanian borders. This characteristic can be attributed to multinational companies.

A sustainable business environment is also notable for its unemployment rate. It is 2.2%, higher than in 2018 by 1.8%, but it remains lower than the national level of 3.4%, ranking the region second place after the Bucharest-Ilfov Region with a 1.2% unemployment rate. This indicator, by its low value, places the Western Region among the best-ranked development areas in this regard for 2020.

From the activity fields point of view, in the West Region in 2020, most enterprises were micro and SMEs. They are located in Timiș and Arad. The main occupational domain was “trade” for 26%; “professional, scientific and technical activities” for 20.2%; “constructions” for 11.0%; and “manufacturing” for 9.4%.

The companies involved in the research are important providers of flexible production, quality, and logistics solutions that cover customers’ real needs. They are active in the automotive industry and in units involved in mass production in the electronics industry, household appliances, and cosmetics industries. In their case, it was noted that to achieve operational objectives such as developing employee skills, using advanced technologies, and exceeding customer expectations, it is important to use innovative methods and tools such as single platforms, which allow access to the most important information from a distance, anywhere, anytime.

Ten enterprises were considered for the survey application. According to current legislation [16], these are classified as large enterprises with more than 250 employees. In all cases, the internal communication before the mentioned period was made using the traditional approach, through face-to-face meetings. The online sessions were exceptions encountered only for the international management board seminars.

Most analyzed corporates use today’s online communication (Internet and Intranet) for different purposes. The main areas are future employee recruitment (websites, recruitment platforms); employee’s skills for permanent development (platforms, smartphone applications); and interaction and cooperation between different departments, including the external environment (ERP Software-Enterprise Resource Planning). These approaches facilitate the information flow between all business functions and manage connections with external stakeholders. The external environment allows customized solutions to the client, traceability, transparency within the production processes, and aspects that impact the quality of their products and services, ensuring online communication between employees, different departments, and management. Throughout the dedicated platforms and advanced databases, the results of the company’s operations are updated for their customers, suppliers, the local community, and the environment.

Many corporations involved in the study have positively embraced environmental protection issues to fulfill their social responsibilities as global citizens. In this sense, they have designed “Environment Philosophy” and promoted ecological protection activities according to the management system of ISO14001 [17].

They provide information, communication, and adequate training of stakeholders to increase the internal organizational and external understanding of commitments and the company’s actions towards the environment, respectively, by setting ecological objectives, monitoring progress, evaluating results, and defining future activities for the continuous improvement of the performance of the company’s initiatives towards durability.

Most multinational companies have allowed us to apply questionnaires in the field and promote values such as competitiveness and innovation based on a global network of knowledge and experience to achieve sustainable, profitable growth and lifelong learning in response to market dynamics and business in general. Other values promoted are a positive attitude, meritocracy, perseverance, and social responsibility, which ensures the balance between the private and professional life of the employees and the company’s management.

The management of the companies included in the research believes that good online communication between employees, different departments, the company’s superiors, and the external environment allows customized solutions to clients and traceability and transparency in production processes and company services. Based on this, the communication principles of the corporation’s management with subordinates and the latter with command are established, with an essential role in substantiating the strategic and operational decisions.

The motivation of the study was also strengthened by other studies recently published, which underline the negative impact of the pandemic situation on the employees. It appears to be an additional stress factor that must be considered going forward [18,19]. Independent of the type, stressors determine an uncertain working environment, and companies must develop strategies to decrease them [20,21]. Such situations affect not only the employee’s direct performance but also their health [22]. The company management had to implement efficient communication strategies to increase the value-added percent of each employee [23].

The research plan was built up based on six working premises. The first one (**H1**) *assumed that the pandemic period COVID-19 influenced the methods of communication in international companies*, a hypothesis that was confirmed after research. In (**H2**), it was *considered that during the pandemic, online connection (e.g., Microsoft Teams, Skype, etc.) was appreciated by the company’s employees*, which was accepted. The third (**H3**) *supposed that face-to-face conversation, telephonic, and e-mail could be ways of communicating appreciated by employees*, an assumption that was rejected by the research. The next one, (**H4**), *presumed that direct contact with co-workers during the COVID-19 pandemic was different from that under normal conditions*. The investigation confirmed the hypothesis. Another premise ascertained was (**H5**). It corroborated information regarding the *communication of superiors with employee’s quality improvement*, and the data collected demonstrated it. The (**H6**) aimed to determine *if the communication staff of multinational employees was difficult during the pandemic.* The affirmation was ascertained.

This article contains five sections. The first one establishes the context of the research, how companies communicate with employees, aspects related to their social responsibility regarding the environment and communication with employees, and the values promoted by them. There are also indicated working hypotheses. The second segment reviews the general theoretical framework of the research. It is followed by interpreting the research data based on the formulated assumptions. The third part describes the research methodology. The conclusions and future perspectives of its scientific demarche are presented in the final segment.

As a novelty, the current research shows that regardless of crises, company employees prefer face-to-face communication, to the detriment of other communication methods.

## 2. Literature Review

Communication is essential in every aspect of life and each type of activity carried out. However, through a brief general analysis, it can be seen that, as is usually the case with those daily aspects that seem so simple, they can often become very complicated. Such an impression is generally determined by the belief that listening is enough for good communication. Overall, this is largely true. Active listening is an essential element in communication. Beyond that, it is necessary to know certain aspects related to the systems of representation and the needs, desires, and aspirations to be communicated, taking into account the interlocutor, the message, and the context in which it is transmitted. The last-mentioned aspect increased recently in significance. The pandemic crisis left its mark deeply on the human interrelationship pattern. COVID-19 determined human interaction and message perception modifications in different areas, such as air travel, sanitary safety, or organization computerization [24,25,26]. The situation was also explored in our case as the starting point in elaborating the first hypothesis.

At a theoretical level, conversation involves transmitting a message from a sender. It has to be coded according to the sender’s knowledge and cultural and educational baggage, sent through an appropriate channel to the receivers, and followed by feedback from them, which is also coded [27,28,29,30,31].

The receiver does not respond directly to the message through the sense organs, i.e., images, sounds, and sensations, but first attaches meanings and symbols to it based on lived experiences. Until then, the message transmitted can be very different from that received [32]. It is important to consider the receiver’s ability to decode the message. These are determined by their educational, cultural, and formative knowledge levels and lived experiences.

Communication channels also have an important role in transmitting messages and can be formal, predetermined, the most used in the entrepreneurial environment, or informal, as is found in friendships. The chosen model may differ depending on the environment, and in certain periods with turbulent external conditions, the choice becomes conditioned by these situations. In the case of companies, it is advisable to determine the employee’s preferences to maximize the communicational effect [33]. The method must also consider the social background [34,35]. Considering these premises and the references currently present in the domain, we formulate the second and third theories to be tested in the survey. Bruggerman et al. presented the impact of online activity on the teaching field [36]. Their findings could be used to improve the educational methodologies further. Moreover, our results can be considered valuable inputs for future action plans to apply in similar conditions.

Communication can be (1) intrapersonal communication (with oneself); (2) interpersonal (with others); (3) metacommunication, or what is transmitted to the interlocutors through gestures, facial expressions, and the intonation of the voice, body posture, clothing, etc. The status in any community is always influenced by the individual’s capacity for emotional sharing. These can be transformed into positive outcomes depending on social, personal, and professional status [37]. A certain level of synchronicity in a co-worker’s gesture may improve their work connections [38].

**Figure 1:**
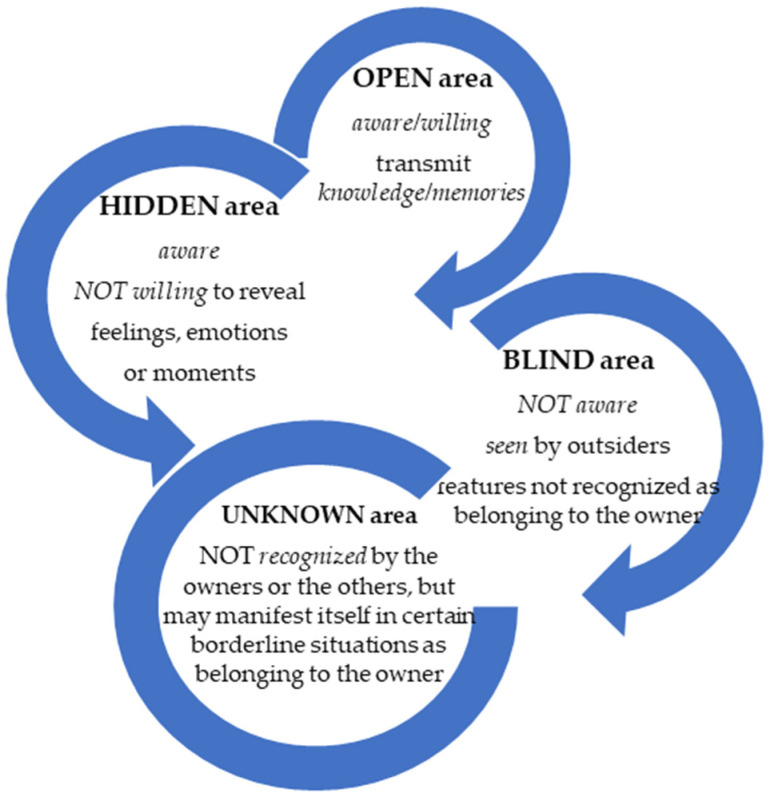
Johari Window model of communication.

According to the Johari Window model [39], each constructive [40] communication results from the four areas of interconnectedness (Figure 1).

**Figure 2:**
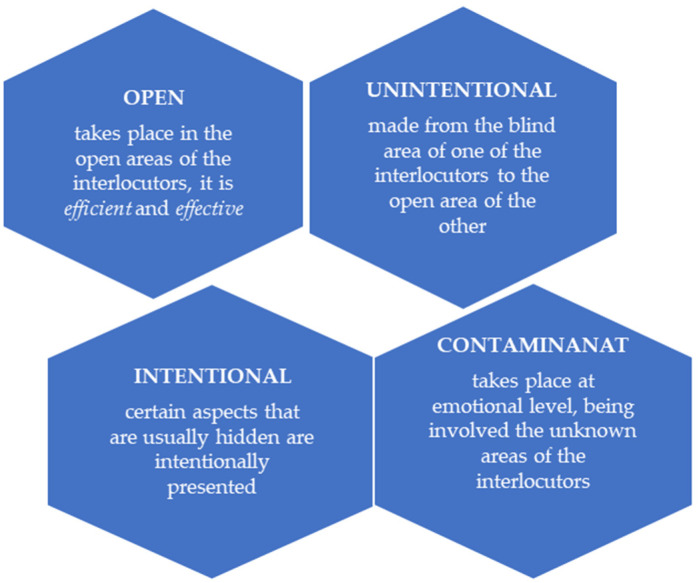
Connection forms.

The combination of the four areas results in similar types of communication, as seen in Figure 2. Unintentional transmission reveals a part of one interlocutor’s personality that they do not intend to show. Such aspects can be used for constructive feedback through guidance and counseling. Throughout intent transmission, self-exposure takes place. The input can be received and given, and it is also an important element for the formation and development of productive relationships development.

It is considered that we need to know how to discipline ourselves to listen. When doing so, it is necessary to pay attention to the words and what is not said, i.e., feelings, reactions, and gestures, and to determine what all this tells us actively. In *The Genius in All of Us* (2010), David Shenk said of active listening that “information overload has replaced the lack of information and become a new political, social and emotional problem”. It is also observed that controlling our internal noise also affects the interlocutor so that they can focus better on the conversational process [41].

The communication process involves direct actions from the partners implicated: hearing, understanding, translating words into meanings, assigning signification to information, and evaluating it. Through these actions, active listening is achieved that supports open conversation. Then, the attention toward the discussion partner increases, the difficulties of understanding is reduced, and the discussion climate improves.

Active listening includes important elements such as mimicry, gestures, the interlocutor’s posture, tone of voice, rhythm, and accent. As a result, the partner offers empathy. The reaction interval is dependent on the context. In a descriptive background, the process tends to be shorter [42]. These principles are applied in different sectors to improve the services offered [43]. Several recent studies underlined the importance of socialization in various industries and media to maintain functional relations during a crisis [44,45,46,47]. These were the basis for postulating the subsequent statements.

## 3. Materials and Methods

The study was made over two years, 2021 and 2022. It is a comparative one and evaluates the quality and methods of communication in international companies. The questionnaire developed includes factual, closed, and semi-open questions, punctual for each element taken in the research, and scaled to queries. The survey was applied using the Google Forms platform. Management and execution staff employed in the corporations from Arad and Timiș counties responded to the survey queries. The sample is representative of the multinationals in the chosen region, given the existing limitations and constraints in this regard. One of the study’s limitations was that it could only be made online due to the COVID-19 pandemic. Only those who wanted to express their opinion responded, not all the companie’s employees.

In the first year, 84 persons answered, and 90 in 2022. With a total of 174 respondents, the research is valid. The number is representative, considering the approximately 5800 people employed in multinationals in the two counties. The standard error was ±5%, with a probability threshold of 95%.

The sampling method applied was non-probabilistic. It consisted of using the statistical step based on a source of personal data, respecting the number/identification code order of the respondents, and being distributed to different people from one year to another. The statistical step was 64. We could not apply a smaller step because some of those included in the sample did not answer affirmatively to our requests for various reasons: afraid of losing their job, fear of providing confidential information about the company, etc.

Out of the total sample, 48.27% were respondents for 2021 and 51.73% for 2022. Of the latter, 46.66% belonged to the management staff of operational/medium level (head of shift, head of a department, head of service/office) and 53.34% to the executive team. As a general request, the enterprises wanted their identities to be protected but agreed with the employee’s interview.

The chi-square tests were applied regarding the descriptive statistics used to study two variables, which focused on extracting information about the sample. The parameters that were considered referred to time on the one hand and the changes related to communication from the telework period, respectively, and the forms of sustainable communication on the other hand. After collecting the information, databases were created in the program SPSS-IBM Statistics-Version 23, provided by IBM Corp.

## 4. Results and Discussion

The research was conducted at two different times of the COVID-19 pandemic crisis. In this regard, in 2021, we had as respondents people with a good representation. Their sociodemographic aspects are included in Table 1.

The respondents from 2021 have different training categories. A high degree of preparation for the respondents can be observed, and a brief analysis highlights a fairly high level of job stability among them. Another aspect specific to the analyzed period was the proportion of total working time in which the interviewees benefited from working from home during the COVID-19 pandemic. Out of the survey, it was confirmed that almost two-thirds of respondents had telematic communication in various forms.

For 2022, the factual data for staff coordination profiles are presented in Table 2.

People with a high level of education have the upper share. An interesting aspect was to find seniority constancy among the employees. The coordination staff declared a higher home working percentage than the implementation ones. Around half of them predominantly fulfilled their professional duties in the work field.

The execution of employees’ anthropometric particularities is described in Table 3.

A rather pronounced difference is also observed in terms of the level of education. Even if half the respondents had a high level of training, there were also those with only a vocational bachelor’s degree. Although they were part of the executive staff, we noticed that most of them had over three years of seniority in work in the current company.

Regarding the COVID-19 pandemic, it can be noted that in 2022, over half of the employees did not use teleworking anymore (58.3%). In 2021, the situation was the opposite. Almost two-thirds (61.9%) of the respondents developed their work preponderantly from home (75–100% of the total duration of a daily job).

The respondents’ training levels also influenced the results obtained in the research. Thus, the ones with higher education had a much more significant share in 2021 than in 2022. In contrast, the current standing was higher in 2022.

**Figure 3:**
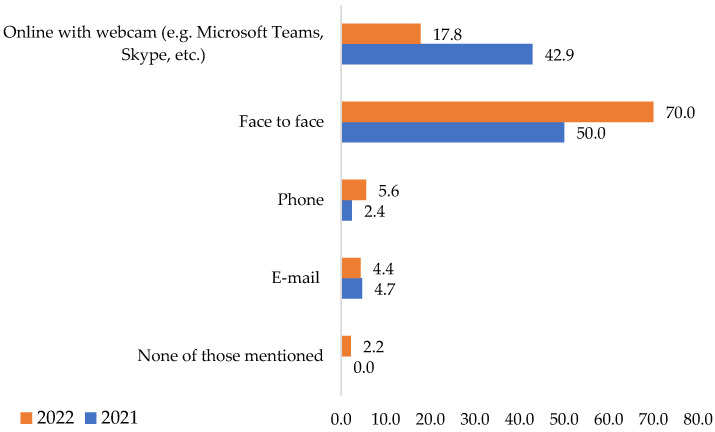
Communication methods preferred in working relationships. Source: Data from field quizzes, 2021, 2022.

Following the data analysis, the results obtained reflect the prevailing situation encountered. Regarding the comparative analysis of preferences over communication methods in the period studied, we can see from Figure 3 that in 2021, 50% of respondents preferred face-to-face communication, and 42.9% preferred online communication. In 2022, the situation was different. Respectively, 70% said they liked face-to-face communication better, and only 17.8% were the online type. These results confirm the first **H1** hypothesis, in which it was assumed that the pandemic period COVID-19 influenced the methods of communication in corporations. By application of the chi-square tests, the *p* obtained is <0.005. The variables are associated, meaning that the COVID-19 pandemic period and the passage of time influenced the respondents.

One possible factor explaining the results obtained is the pandemic situation itself. After 8 March 2022, in Romania, restrictions were lifted. Therefore, the companies’ employees could benefit from their return to activity that was close to normal. In such a situation, 70% of the interviewees preferred face-to-face communication.

Online communication with co-worker’s satisfaction degrees was also investigated. In 2021, 44% of them sustained that they were content using software such as Microsoft Teams, Skype, etc. By contrast, in 2022, the percentage decreased to 14.4%. The rate of staff dissatisfied with this communication method was 15.6% but accepted it. In 2021, only 4.8% had the same opinion.

**Figure 4:**
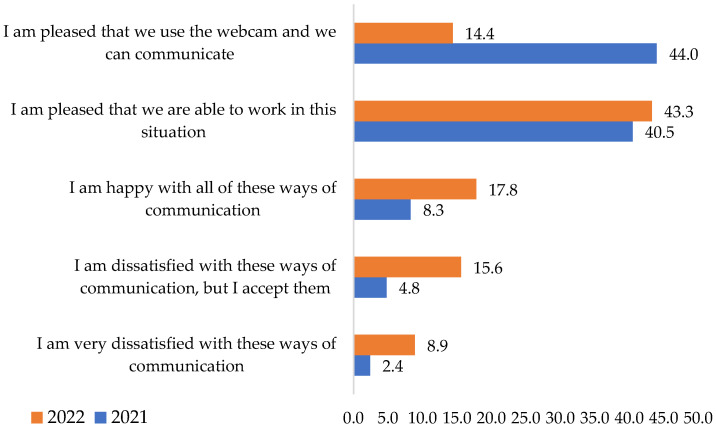
The satisfaction/dissatisfaction degree in the online communication with colleagues (e.g., Microsoft Teams, Skype, etc.). Source: Data from field quizzes, 2021, 2022.

As seen from the research data results, **H2** is also confirmed (Figure 4). It assumed that during the COVID-19 pandemic, online communication (using Microsoft Teams, Skype, etc.) was a mode chosen by multinational companies and appreciated by employees.

Following the application of chi-square tests for this question, it results that the variables are associated. The structure of the answers was different over the two years. Considering also that aspect, the hypothesis from which **H2** started was confirmed (Table 4).

The comparative data analysis shows that the majority preferred face-to-face communication. The percentage is almost the same in the two years (36.9% in 2021 and 41.1% in 2022). The opportunity to observe non-verbal communication (gestures, body communication, clothing, etc.) motivates this choice. Around 22% of the interviewees considered that they could only perceive emotions and feelings in this way (2022).

**Figure 5:**
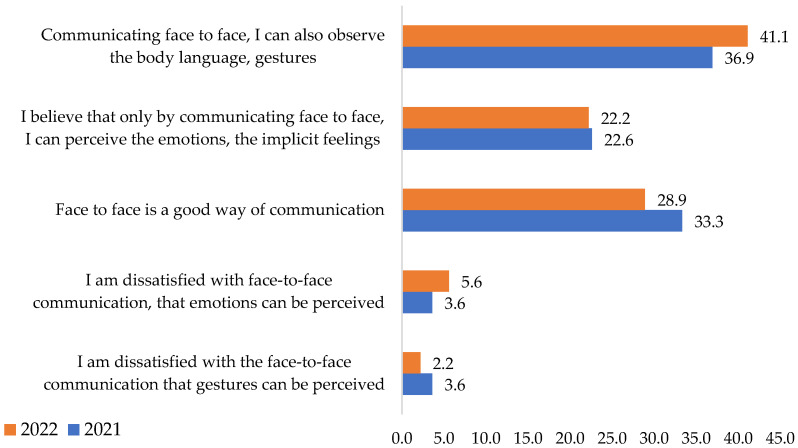
Respondents’ preferences regarding face-to-face communication. Source: Data from field quizzes, 2021, 2022.

The percentages of those dissatisfied with face-to-face communication were also close in the analyzed period, respectively, 3.6% in 2021 and 5.6% in 2022. The respondents claimed they were disappointed because their emotions could be perceived in such a situation (Figure 5).

The third hypothesis (**H3**) was rejected. It was assumed that during the COVID-19 pandemic, face-to-face communication, telephone communication, and e-mail could be methods appreciated by employees.

The applied statistical test shows a *p* > 0.005, indicating that the variables are not associated, with respondents choosing similar answers. Therefore, the difference between their answers over the two years does not have a relevant statistical significance, so part of hypothesis **H3** that of preference for face-to-face communication, is not checked (Table 5). In other words, regardless of the existing situation, face-to-face communication was the preferred one. The pandemic did not influence it.

**Figure 6:**
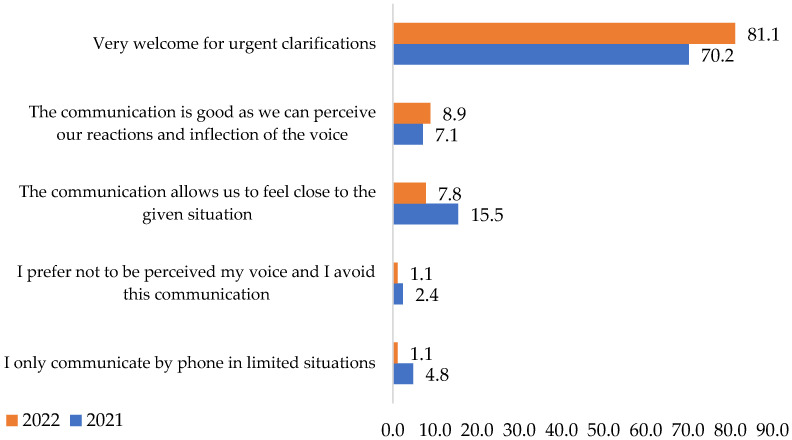
Telephone communication with co-workers. Source: Data from field quizzes, 2021, 2022.

Telephone communication with co-workers was considered helpful for urgent clarifications by 70.2% of respondents in 2021 and by 81.1% of them in 2022. In 2021, only 15.5% of answerers regarded it as a way that allowed them to feel connected during the pandemic. However, the percentage decreases in 2022 to 7.8%., Figure 6.

The chi-square test shows that the variables are not associated, and the differences between the answers do not have statistical significance. Following the evaluation carried out in this regard, we can say that the telephone, as a means of communication, is used in the work environment more and more for urgent clarifications. That is the motivation for which this part of the **H3** hypothesis is also rejected.

**Figure 7:**
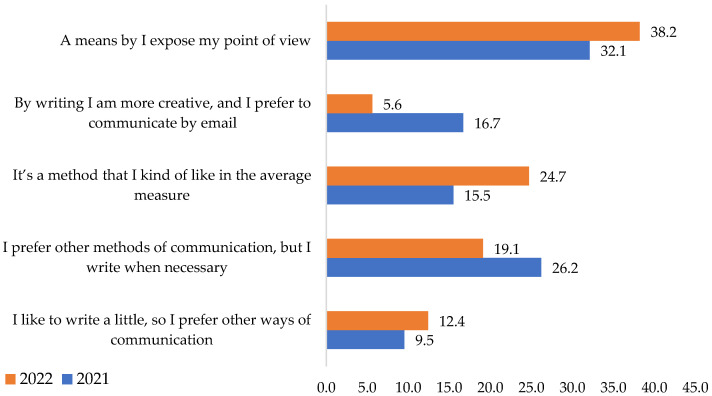
E-mail conversation with co-workers. Source: Data from field quizzes, 2021, 2022.

From the research, it can be seen that e-mail communication was considered a method by which respondents broadly expressed their views. Around one-quarter of them used it only when necessary (Figure 7). The perspective is imparted by 32.1% in 2021 and 38.2% in 2022.

The chi-square test shows that the variables are not associated. Moreover, this part of the **H3** hypothesis is rejected. Opinions on e-mail communication remained unchanged during the two years.

**Figure 8:**
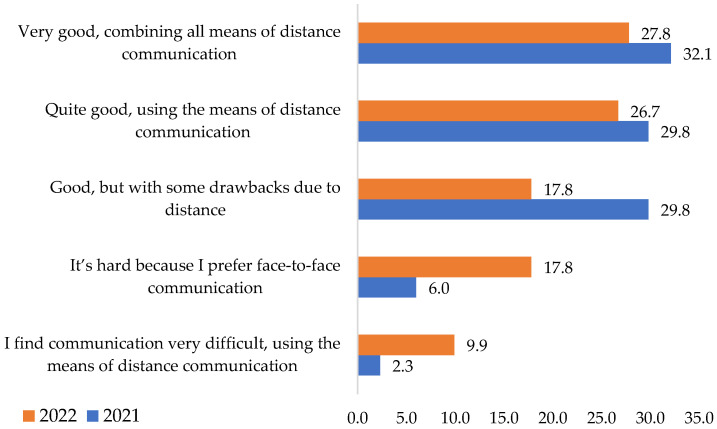
Co-worker’s connection during the pandemic. Source: Data from field quizzes, 2021, 2022.

During the study period, 32.1% of respondents for 2021 and 27.8% for 2022 considered direct communication with colleagues very good, combining all means of distance communication. A proportion of 29.8% in 2021 and 26.7% in 2022 regarded it as quite good, using the distance instruments of communication. Only 6% in 2021 and 17.8% in 2022 said it was difficult, preferring face-to-face communication, as shown in Figure 8.

Conversation with co-workers remains an important issue for respondents. The research results confirmed the **H4** hypothesis that during the COVID-19 period, direct communication with co-workers was different from that under normal conditions. In this case, the two variables were associated. It can be said that direct contact in labor relations is better without social distance.

**Figure 9:**
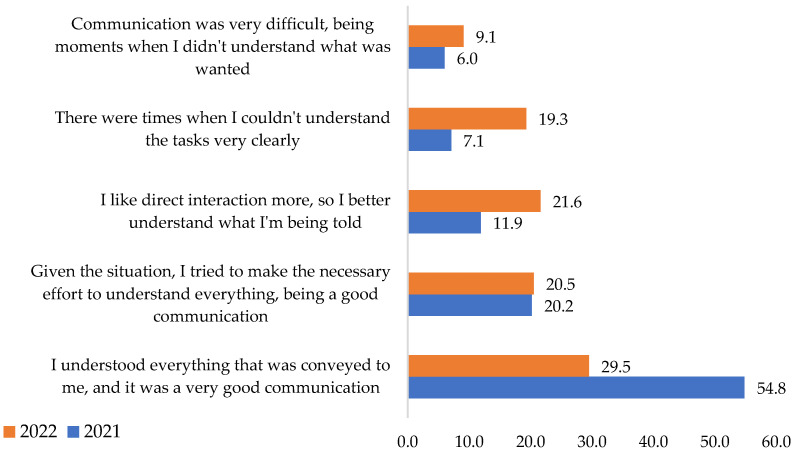
Staff worker’s quality connection. Source: Data from field quizzes, 2021, 2022.

Regarding the communication quality with superiors, 54.8% of respondents from 2021 understood everything that was sent to them and had a very good conveyance. In the next year, the percentage decreased by almost half (Figure 9). According to the results, the leader’s employee link in the pandemic was better. The finding confirms the **H5** hypothesis.

The superior’s employee communication in 2021 was much better than the general average of the analyzed period. The results are due to managers’ greater interest and special attention to the quality of communication. These aspects highlight each company’s organizational culture and communication with stakeholders, including its employees and the external environment.

The chi-square test application indicates the association of the variables. As a result, the **H5** hypothesis is confirmed, showing that there was a change in their opinion.

**Figure 10:**
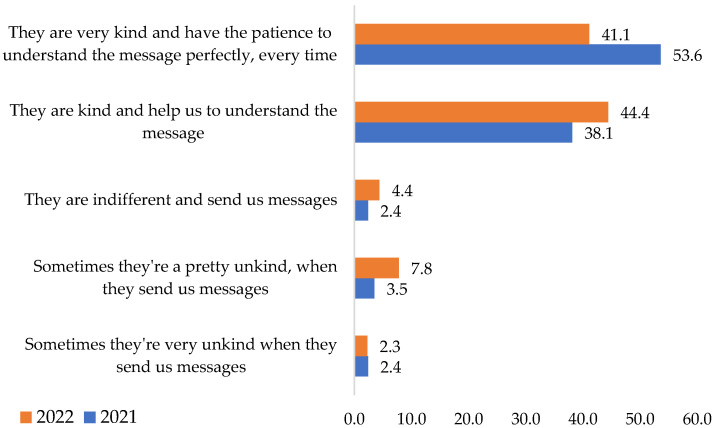
Leaders and staff communicating attitude: Data from field quizzes, 2021, 2022.

In support of the above (including **H5**) comes the following question that shows the kindness of superiors in communicating with employees. Thus, the respondents considered the seniors very kind and patient and perfectly understood the message sent each time, according to 53.6% in 2021 and 41.1% in 2022 (Figure 10). The importance of the superior’s involvement in ensuring the best communication framework was also underlined by Jämsen et al. [48].

**Figure 11:**
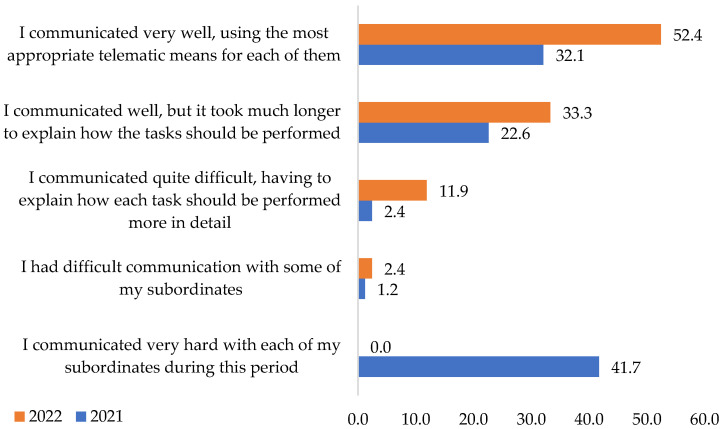
Communication with subordinates during the pandemic: Data from field quizzes, 2021, 2022.

In this research, we also considered the staff opinions of the analyzed companies, not only their employees. Thus, they were asked about their opinion regarding communication with subordinates during the pandemic. “*I communicated very hard with each of my subsidiaries*” during this period is the statement supported by 41.7% of respondents for 2021 (Figure 11). In an assertion not backed by respondents in 2022, no coordinator considered that they did not encounter difficulties communicating with employees under normal conditions. These sincere staff opinions regarding the efforts made during the pandemic in communicating with employees confirm hypothesis **H6**. This assumes that the connection between the staff multinationals and their employees was much more difficult during the COVID-19 pandemic.

The staff considered that they connected very well using the most appropriate telematic means for each employee in proportion to the affirmative answers of 32.1% in 2021 and 52.4% in 2022, followed by those who considered that they communicated well. Still, they involved more time explaining the tasks to be performed in the proportion of 22.6% in 2021 and 33.3% in 2022.

Applying the chi-square test confirms the association of the variables. It results from the correlation of the employees’ answers with the staff that the last ones performed more intense activity during the considered period. The team effort had a positive appreciation of employees regarding communication and a good understanding of tasks to be accomplished. Therefore, hypothesis **H6** is confirmed.

## 5. Conclusions

The multinationals from the Romanian Western Region had a sustained evolution in the last twelve years with 4.2%. They were surpassed only by micro-enterprises which registered an increase of 26% in the same period. The polarization is in Timiș and Arad counties, with 70.35% of the region’s employees.

These aspects are also confirmed by the employee’s distribution analysis of the size classes of companies. A study indicates an individualization of the West Region. One-third of employees work in large enterprises. A proportion similar to the distribution by size classes is in the Bucharest-Ilfov Region, the country’s most developed region.

Specific to Romania and other developing countries is that these multinationals largely ensure productivity and economic well-being, according to a series of variables in Structural Business Statistics (SBS). The fulfillment of the set of indicators of Sustainable Development Indicators at the Territorial level for Romania (SDIT) characterizes a local, durable development. One important factor in any business evolution is and will be conditioned by good communication [49]. Other aspects that also need to be considered are the methods used and the audience particularities [50].

Like other studies in the area [51], ours also underlines the pandemic’s impact on economic and relational domains. In this context, the focus of research on the preferences of employees in multinational companies from the Western Region, the ways of communication over the telework period, and the forms of sustainable communication during the particular context is justified and timely. The following conclusions resulted after compiling data:

- Due to the drastic restrictions imposed by the authorities following the COVID-19 pandemic, the management of most multinational companies in Western Romania decided that the employees should work from home. After the relaxation of restrictions (8 March 2022), the corporate decision-makers agreed to return to classical status;
- During the telework period, distance communication use patterns (e.g., Microsoft Teams, Skype, etc.) were chosen by the company’s administration and appreciated by employees only in extreme cases (COVID-19 pandemic period). These are used to carry out the activity in the given situation and do not drastically change the classical, in-person approach;
- Regardless of the current status, company employees prefer face-to-face communication. The crisis did not influence this. This form of communication is primarily chosen because it provides an open area of interpersonal interaction. The participants observe non-verbal message transmissions (gestures, body communication, clothing etc.) or can perceive emotions and feelings. Their personality can be identified through unintentional contact to obtain constructive feedback through guidance and counseling. Moreover, it can be formed and develop productive, intentional connections. In this sense, stakeholders’ efficient and effective open dialogs will be encouraged;
- The telephone, as a form of data transmission and connection, is used in the working environment of multinationals more and more only for urgent clarifications, and e-mail only when necessary (opinions on this issue remained constant throughout the research period);
- Direct communicating with co-workers remains an important issue, which is why, regardless of the methods used, it continued to be convenient, good, and differently addressed during the COVID-19 pandemic. Independent of the telematic methods chosen, they turned out to be good. However, direct contact is the most appropriate in labor relations within multinationals;
- An open dialog highlights the organizational culture of each company. It visualizes the care of each one towards the image and the conversation with the stakeholders. The employees and external environment are also considered. Looking over the difficult times that companies experience, including the pandemic, the quality of communication between the management and the employees is much better. It was observed that after March 2022, the connections worsened (the approach was very difficult, including moments when the workers sustained that they didn’t understand their tasks. The effort made by the leadership to optimize the working media strengthens the bilateral engagement in the companies [52].
- Staff and employee connection from corporations was much more difficult during the COVID-19 pandemic. It involved the sustained transmission activity of management. However, the staff considered communicating well, using the most appropriate telematic means for each worker. The time involved in explaining the tasks was longer. The sustained effort was reflected in the positive appreciation of employees regarding the conversations.

Professional communication must be interactive, more proactive, and multi-channel in the new economic and social context (pandemic-related, but also highly computerized, interconnected to transmission worldwide). Thus, in crisis times (such as the COVID-19 pandemic), by necessitating that the vast majority of employees work from home (especially during the restrictions imposed by the authorities, March 2020–2022), the corporate management in Western Romania exploited and learned to use the direct transmission model. It was adapted to distance through the varied methods of online ways (e.g., Microsoft Teams Skype, etc.) made available by management. This contact model was suitable for employees, especially between (superior) managers and workers. An aspect that needs to be underlined is that managers paid more interest and attention to the quality of dispatch with employees (clarity, empathy, patience). These skills and competencies had to be learned or re-updated in certain situations.

Moreover, it is recommended that employees take advanced training courses in remote communication. However, the connection that is carried out mainly face to face remains the most requested mode by employees, which helps to rebuild the organizational culture and the solidity of the company affected by the COVID-19 pandemic, and it remains appreciated by those surveyed. Regarding other means of contact used by multinationals (the telephone/mobile phone or e-mail), it was observed that their marginal use has a smaller contribution to the efficiency of connection within the Romanian multinationals in the West.

A multinational company or a solid subsidiary in an emerging country/region must ensure sustainability as a concern of today’s society to achieve a balance between security and social cohesion, economic performance, and environmental protection to guarantee the next generation’s future [53].

It is recommended that the coordination teams continue to pay attention to the communication methods and relationship with the employee’s training and education considering these indications. Such an attitude will contribute to companies and, generally, to the evolution of a stable society [54].

Moreover, all forms of schooling are very important, both the professional ones, the high school and the university training, all of which should include these essential elements regarding the analyzed topic. Coaching must pursue employee statuses, aptitudes, and priorities [55]. If the online conversation is something natural for the young generation, in the case of the more mature, the situation could represent a stress factor. It can determine an unfavorable attitude toward work in all aspects [56]. One of the projects developed by some authors referred to aspects and methods of communication and the development of soft skills in the continuous framework. STRATAGAME is the Strategic Partnership For Soft Skills Building Through Gamification (https://stratagame.erasmus.site/ro/ (accessed on 20 June 2022)).

One objective of the current research is to highlight the importance of communicatiog methods in companies in general and to develop joint projects with the business environment on these topics. The necessity of such a research approach is also sustained by other studies that have been recently published. These underline the importance of implementing actions that support a performant background based on adjustability principles [57].

We also want to point out some aspects related to the sustainability and durability over time of our research:

- The research results can be a tool to understand/support employees/employers and in other similar situations with an impact on economic and social activities);
- The study can be used as informational support for the business environment and local authorities through the conclusions drawn;
- The COVID-19 pandemic is a life situation that can be learned from. The information collected, processed, and interpreted in the paper can represent a source for developing a good practice code in health situations of crisis level or otherwise. The data obtained could be considered a base for communication strategies’ improvement and/or development in different institutions during considered cases. In their study, Zhou et al. mentioned the discrepancies between academics and the public in limiting conditions [58]. It is important to find and/or project from ground bases instructive support for all actors involved in daily activities, especially if we consider exceptional situations [59].

## Figures and Tables

**Table 1:** Sociodemographic characteristics of the execution staff from 2021.

Features	Characteristics	Share (%)
Age	18–30	20.1
	31–40	43.7
	41–50	23.3
	51–60	12.9
Gender	Male	45.2
	Female	54.8
Education level	High school education	1.2
	University degree	59.5
	Master degree	35.7
	PhD	3.6
Work experience in years	Under 1	13.1
	1–3	25.0
	3–5	21.4
	5–7	16.7
	Over 7	23.8
Percent of telework from the total time realized	Under 25	10.7
	25–50	17.9
	50–75	9.5
	75–100	61.9

**Table 2:** Sociodemographic characteristics of the management staff from 2022.

Features	Characteristics	Share (%)
Age	18–30	23.8
	31–40	45.2
	41–50	26.2
	51–60	4.8
Gender	Male	40.5
	Female	59.5
Education level	Vocational school	-
	High school	23.8
	Post-secondary	4.8
	Higher education	71.4
Work experience in years	Under 1	11.9
	1–3	11.9
	3–5	14.3
	5–7	26.2
	Over 7	35.7
Percent of telework from the total time realized	Under 25	52.4%
	25–50	-
	50–75	-
	75–100	21.4%

**Table 3:** Sociodemographic particularities of the execution staff from 2022.

Features	Characteristics	Share (%)
Age	20–30	42.6
	31–40	27.7
	41–50	25.4
	51–60	4.3
Gender	Male	43.7
	Female	54.2
	Undefined	2.1
Education level	Vocational school	10.4
	High school	39.6
	Post-secondary	-
	Higher education	50.0
Work experience in years	Under 1	18.8
	1–3	18.8
	3–5	27.1
	5–7	14.6
	Over 7	20.7
Percent of telework from the total time realized	Under 25	58.3
	25–50	16.7
	50–75	16.7
	75–100	8.3

**Table 4:** Chi-square tests for the elements that meet the expectations with online communication in relationships with colleagues (e.g., Microsoft Teams, Skype, etc.).

	Value	df	Asymptotic Significance (2-Sided)
Pearson chi-square	24.362 ^a^	4	0.000
Likelihood ratio	25.501	4	0.000
Linear-by-linear association	21.346	1	0.000
N of valid cases	174		

^a^ 0 cells (0.0%) have an expected count of less than 5.

**Table 5:** Chi-square tests for the face-to-face communication preference.

	Value	df	Asymptotic Significance (2-Sided)
Pearson chi-square	1.124 ^a^	4	0.891
Likelihood ratio	1.130	4	0.890
Linear-by-linear association	0.290	1	0.590
N of valid cases	174		

^a^ 0 cells (0.0%) have an expected count of less than 5.

## Data Availability

Not applicable.
